# Duration of SARS-CoV-2 Immune Responses Up to Six Months Following Homologous or Heterologous Primary Immunization with ChAdOx1 nCoV-19 and BNT162b2 mRNA Vaccines

**DOI:** 10.3390/vaccines10030359

**Published:** 2022-02-24

**Authors:** Ulrika Marking, Sebastian Havervall, Nina Greilert-Norin, Henry Ng, Kim Blom, Peter Nilsson, Mia Phillipson, Sophia Hober, Charlotta Nilsson, Sara Mangsbo, Wanda Christ, Jonas Klingström, Max Gordon, Mikael Åberg, Charlotte Thålin

**Affiliations:** 1 Department of Clinical Sciences, Karolinska Institutet Danderyd Hospital, 182 88 Stockholm, Sweden; ulrika.marking@ki.se (U.M.); sebastian.havervall@ki.se (S.H.); nina.greilert@ki.se (N.G.-N.); henry.ng@mcb.uu.se (H.N.); kim.blom@folkhalsomyndigheten.se (K.B.); max.gordon@ki.se (M.G.); 2 Department of Medical Cell Biology, Uppsala University, 751 23 Uppsala, Sweden; mia.phillipson@mcb.uu.se; 3 Department of Microbiology, Public Health Agency of Sweden, 171 82 Solna, Sweden; charlotta.nilsson@ki.se (C.N.); jonas.klingstrom@ki.se (J.K.); 4 Department of Protein Science, KTH Royal Institute of Technology, SciLifeLab, 114 28 Stockholm, Sweden; peter.nilsson@scilifelab.se (P.N.); sophia@kth.se (S.H.); 5 Division of Clinical Microbiology, Department of Laboratory Medicine, Karolinska Institutet, 171 76 Stockholm, Sweden; 6 Department of Pharmacy, SciLifeLab, Uppsala University, 751 23 Uppsala, Sweden; sara.mangsbo@farmaci.uu.se; 7 Center for Infectious Medicine, Department of Medicine Huddinge, Karolinska Institutet, 141 52 Stockholm, Sweden; wanda.christ@ki.se; 8 Department of Medical Sciences, Clinical Chemistry, SciLifeLab, Uppsala University, 753 09 Uppsala, Sweden; mikael.aberg@medsci.uu.se

**Keywords:** COVID-19, SARS-CoV-2, SARS-CoV-2 vaccination, heterologous, immune response, duration, antibodies, humoral response, T-cells, immunity, immunology

## Abstract

Heterologous primary immunization against SARS-CoV-2 is part of applied recommendations. However, little is known about duration of immune responses after heterologous vaccine regimens. To evaluate duration of immune responses after primary vaccination with homologous adeno-vectored ChAdOx1 nCoV-19 vaccine (ChAd) or heterologous ChAd/BNT162b2 mRNA vaccine (BNT), anti-spike-IgG and SARS-CoV-2 VOC-neutralizing antibody responses were measured in 354 healthcare workers (HCW) at 2 weeks, 3 months, 5 months and 6 months after the second vaccine dose. T-cell responses were investigated using a whole blood interferon gamma (IFN-γ) release assay 2 weeks and 3 months post second vaccine dose. Two hundred and ten HCW immunized with homologous BNT were enrolled for comparison of antibody responses. In study participants naïve to SARS-CoV-2 prior to vaccination, heterologous ChAd/BNT resulted in 6-fold higher peak anti-spike IgG antibody titers compared to homologous ChAd vaccination. The half-life of antibody titers was 3.1 months (95% CI 2.8–3.6) following homologous ChAd vaccination and 1.9 months (95% CI 1.7–2.1) after heterologous vaccination, reducing the GMT difference between the groups to 3-fold 6 months post vaccination. Peak T-cell responses were stronger in ChAd/BNT vaccinees, but no significant difference was observed 3 months post vaccination. SARS-CoV-2 infection prior to vaccination resulted in substantially higher peak GMTs and IFN-γ levels and enhanced SARS-CoV-2 specific antibody and T cell responses over time. Heterologous primary SARS-CoV-2 immunization with ChAd and BNT elicits a stronger initial immune response compared to homologous vaccination with ChAd. However, although the differences in humoral responses remain over 6 months, the difference in SARS-CoV-2 specific T cell responses are no longer significant three months after vaccination.

## 1. Introduction

In the global struggle to achieve and maintain efficient COVID-19 vaccine programs, heterologous vaccine regimens attract great interest [1,2,3,4,5,6,7]. With tolerable safety [1,8,9], and both immunogenicity [2,3,4,5,6,7,8,10,11,12,13,14] and effectiveness [15,16,17,18,19,20] exceeding that of homologous adenovector immunization, heterologous primary vaccination is as of 7 December 2021 recommended by European Medical Agency and European Centre for Disease prevention and Control [21]. However, more data on the duration of immune responses following heterologous primary vaccine protocols are needed [21,22].

In this study, T-cell responses against SARS-CoV-2 wild type and antibody responses against SARS-CoV-2 wild type and four variants of concern (VOC: Alpha, Beta, Gamma, and Delta), were analyzed over six months after two doses adeno-vectored ChAdOx1 nCoV-19 vaccine (ChAd) or one dose ChAd followed by one dose BNT162b2 mRNA vaccine (BNT) in 354 healthcare workers (HCW) with and without SARS-CoV-2 infection prior to vaccination. Healthcare workers vaccinated with two doses BNT (*n* = 210) were enrolled for comparison of VOC-neutralizing responses.

## 2. Materials and Methods

The COMMUNITY study is an ongoing longitudinal cohort study including 2149 HCW [23,24]. Blood samples were first obtained at study inclusion in April 2020 and are from then on collected prospectively at a minimum of four months interval. Clinical data including symptomatology is collected at every follow-up through a smart phone application-based questionnaire. SARS-CoV-2 spike-specific IgG responses were analyzed by multiplex antigen bead array at every follow-up preceding vaccination (FlexMap3D, Luminex Corp, Austin, USA) as described elsewhere [23,24].

HCW received vaccination with either BNT or ChAd starting January and February 2021, respectively. Upon reports on vaccine induced thrombocytopenia and thrombosis [25], the use of adenovector vaccines was discontinued in persons under 65 years in Sweden. ChAd primed HCW were, as in several other European countries [1], recommended a mRNA vaccine dose but a second ChAd dose at the same dose interval remained an eligible option.

Participants that had received a first dose of ChAd were included in this sub study (*n* = 354). Samples were collected at 2 weeks (*n* = 190), 3 months (*n* = 337), 5 months (*n* = 102) and 6 months (*n* = 92) after second vaccine dose (ChAd or BNT), Figure S1. Participants were stratified by occurrence of SARS-CoV-2 infection prior to vaccination, defined by either a positive PCR test in the national registry holding all PCR-verified SARS-CoV-2 infections in Sweden [26] or a positive anti-spike IgG result at any of the study follow-ups prior to vaccination. Participants with negative results at every follow-up and no documented PCR positivity were considered SARS-CoV-2 naïve. Participants with breakthrough infections, defined as either positive PCR in the national registry or by a non-vaccine induced antibody increase of >2 SD between samplings (*n* = 6) were censored upon detection of breakthrough infection. Of the 354 study participants, 113 (median age 50 (range 22–68) years, 88% women, 40 SARS-CoV-2 recovered) received homologous ChAd vaccination with a median dose interval of 83 (range 70–98) days. Two hundred and forty-one study participants (median age 48 (range 20–69), 88% women, 67 SARS-CoV-2 recovered) received heterologous vaccination with ChAd followed by BNT, with a median dose interval of 91 (range 70–98) days, Table 1. At sampling 2 weeks post second vaccine dose participants answered a standardized questionnaire (Table S3) concerning reactogenicity following their second vaccine dose. Serum aliquots from 210 study participants (median age 50 (range 21–68) years) immunized with two doses of BNT with a 21-day interval (median 21 days, range 21–28 days) and seronegative before vaccination were collected 3 months after second dose.

###  2.1. Humoral Immune Response

Spike-specific IgG against SARS-CoV-2 wild type and surrogate virus neutralization (the ability of antibodies to block the binding of ACE2 to it cognate ligands) against wild type and variants B.1.1.7 (Alpha), B.1.351 (Beta), P1 (Gamma) and 1.617.2 (Delta) were measured using the V-PLEX SARS-CoV-2 Panel 13 for IgG and ACE2 (Meso Scale Diagnostics (MSD), USA. Cat no. K15463U and K15466U) using a similar approach as previously described [27]. Reference Standard 1 in the serology assay is calibrated against the WHO International Standard (NIBSC code: 20/136) and IgG titers are reported in International Units (BAU/mL). Sample dilution for the surrogate neutralization assay was 1:100 and the data are displayed as MSD Arbitrary Units/mL (AU/mL).

### 2.2. Live-Virus Microneutralization

A subset of 17 samples collected 18–21 days (median 20) following homologous ChAd and 17 samples collected 18–20 days (median 19) following heterologous ChAd/BNT (all 34 individuals naïve before vaccine) were analysed using a live-virus microneutralization assay based on cytopathic effects (CPE) with SARS-CoV-2 wild type and Delta strains as previously described [28]. Serum was 3-fold serially diluted. Each dilution was subsequently mixed with equal volume of 4000 TCID50/mL virus (50 μL serum plus 50 μL virus), incubated for 1 h and finally added, in duplicates, to confluent Vero E6 cells in 96-well plates. Original SARS-CoV-2 (severe acute respiratory syndrome coronavirus 2 isolate SARS-CoV-2/human/SWE/01/2020, GenBank: MT093571.1) and the Delta variant (from Statens Serum Institut, Copenhagen, Denmark), were used. Cells were inspected for CPE by optical microscopy. For both wild type and Delta strain, each well was scored as either neutralizing (if no signs of CPE was observed) or non-neutralizing (if any CPE was observed) after 5 days of incubation. The arithmetic mean neutralization titer of the reciprocals of the highest neutralizing dilutions from the two duplicates for each sample was then calculated.

### 2.3. T-Cell Responses

T-cell responses were assessed 2 weeks (*n* = 190) and 3 months (*n* = 337) post second vaccine dose. Whole blood was stimulated with an in-house generated peptide pool containing in total 16 peptides based on the spike, nucleocapsid, membrane and open reading frame 3 and 7 proteins [29,30]. The peptides have an estimated HLA coverage of 97% (class I and II combined) and have shown to provide a specificity of 96.1% when using it as a read-out for specific cellular responses against SARS-CoV-2 [29,30]. IFN-γ levels were determined using the V-PLEX Plus Human IFN-γ Kit (MSD, Rockville, MD, USA).

### 2.4. Statistics

A mixed-effects linear regression model by vaccine regimen was generated for each outcome with adjustment for sex, age, vaccine dose interval and occurrence of SARS-CoV-2 infection prior to vaccination (infection status). Interactions were applied between sex and age, dose interval and infection status with a three-way interaction for vaccine combination, infection status and time since vaccination. Non-linearity was evaluated using ANOVA in nested models and modelled using natural splines where the number of knots was decided by optimal AIC-value.

Risk of side effects were evaluated using a logistic regression model by vaccine type and infection status, interaction adjusted for age and sex with *p*-values from Fisher’s test.

Antibody response was transformed using the log_2_ function. Estimate comparisons are provided as multiplication factors and presented with 95% confidence intervals (CI). The effect on antibody titers by side effects was measured using a linear regression model for the first measured antibody titer similarly adjusted with the addition of time since vaccination. Analyses were performed in statistical program R version 4.1.2 with the nlme-package version 3.1.152.

To minimize interference of variation of time between second dose and sampling when comparing VOC-neutralizing antibody titers three months post vaccination, a sub-cohort of samples collected 3 months (75–105 days) post second vaccine dose was established.

Estimates of correlation between assays were obtained with Spearman’s rank correlation using GraphPad PRISM version 9.2.0. For comparison of VOC neutralizing geometric mean titers (GMTs) Wilcoxson rank-sum test was used (GraphPad, San Diego, CA, USA).

The study complies with the declaration of Helsinki and was approved by the Swedish Ethical Review Authority (dnr 2020-01653). Informed written consent was obtained from all study participants.

## 3. Results

### 3.1. Antibody Titers Up to Six Months after Primary Immunization with Heterologous ChAd/BNT or Homologous ChAd

**Figure 1:**
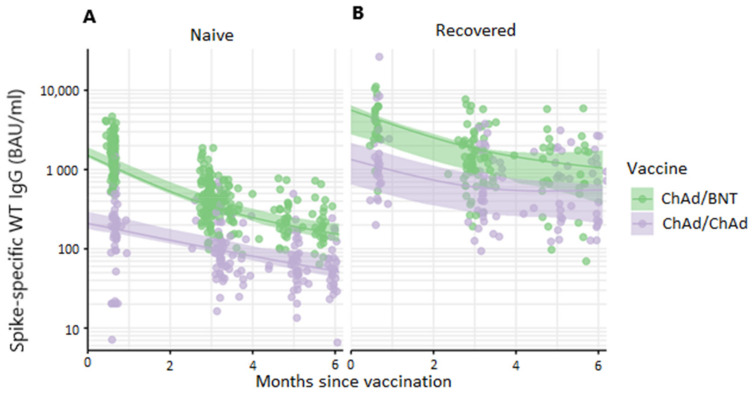
Change in anti-spike IgG titers over 6 months following vaccination. Circles represent individual values, lines represent geometric mean titer with 95% CI in (**A**) participants SARS-CoV-2 naïve prior to vaccination and (**B**) participants SARS-CoV-2 recovered prior to vaccination. ChAd; ChAdOx1 nCoV-19 vaccine, BNT; BNT162b2 mRNA vaccine.

Adjusted for age, sex, vaccine dose interval and time between second vaccine dose and sampling, heterologous immunization with ChAd/BNT resulted in 6-fold (95% CI 4.8–7.6) higher peak anti-spike (WT) IgG GMT compared to homologous ChAd vaccination in individuals naïve to SARS-CoV-2 prior to vaccination, (Figure 1A). As expected, antibody titers declined over time. Half-life of anti-spike IgG was longer following homologous ChAd vaccination (3.1 months (95% CI 2.8–3.6)) as compared to heterologous ChAd/BNT vaccination (1.9 months (95% CI 1.7–2.1)) in SARS-CoV-2 naïve participants **(**Table 2). Consequently, after 6 months the difference in anti-spike IgG GMT was less pronounced with 3-fold (95% CI 2.1–3.8) higher titers after heterologous as compared to homologous vaccination (Figure 1A).

SARS-CoV-2 recovered participants reached substantially higher peak anti-spike IgG GMTs following both vaccine regimens (Figure 1A,B and Table S1). Half-life was increased, although not significantly, after homologous ChAd vaccination (3.8 months (95% CI 3.1–4.7) compared to 2.7 months (95% CI 2.3–3.3) after heterologous ChAd/BNT). At 6 months post vaccination, SARS-CoV-2 recovered participants displayed 6-fold (95% CI 4.4–8.9) and 5-fold (95% CI 3.7–7.3) higher anti-spike IgG GMT after homologous ChAd and ChAd/BNT, respectively, compared to SARS-CoV-2 naïve participants given the same vaccine regimen (Table S1). Antibody decay estimates based on sampling 1–3, 3–6 or 1–6 months are presented in Table 2.

There were no significant dose interval dependent effects on peak serological responses. However, there was an association between shorter dose interval and lower antibody titers at 3 months post vaccination among participants who were SARS-CoV-2 naïve prior to vaccination, diminishing at approximately 80 days dose interval.

### 3.2. Neutralizing Antibody Responses

**Figure 2:**
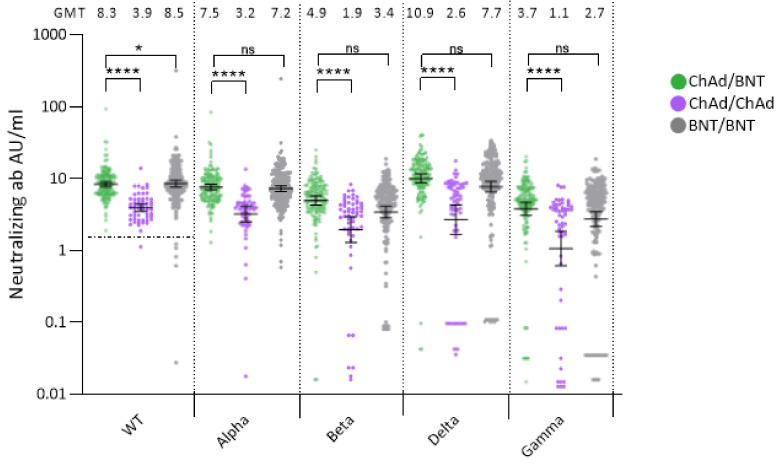
Surrogate virus neutralization GMT 3 months post heterologous ChAd/BNT (*n* = 149, median time to sampling 90 days (range 82–105)) and homologous ChAd/ChAd (*n* = 56, median time to sampling 98 days (range 77–105)) primary vaccination in SARS-CoV-2 naïve participants. Samples 3 months post homologous BNT/BNT (*n* = 210, median time to sampling 87 days (range 75–104)) are presented for reference. Dotted line (wt) represents cut-off value set by the manufacturer. * *p* = 0.0252, **** *p* < 0.001, ns; not significant. Ab; antibodies, WT; wild type, AU; Arbitrary Units, ChAd; ChAdOx1 nCoV-19 vaccine, BNT; BNT162b2 mRNA vaccine.

Surrogate virus neutralization assay was performed in 205 participants sampled three months post second dose. In participants naïve to SARS-CoV-2 prior to vaccination, GMTs of neutralizing antibodies against all four VOCs’ spike were significantly higher following heterologous ChAd/BNT vaccination as compared to homologous ChAd vaccination (Figure 2). Three months after vaccination, BNT immunization resulted in a significantly stronger neutralizing capacity against wild type (*p* = 0.025), but not against the VOCs, as compared to heterologous ChAd/BNT.

GMT of neutralizing antibodies among participants with SARS-CoV-2 infection prior to vaccination exceeded those in naïve participants, regardless of vaccine regimen (Figure S2).

A live-virus microneutralization assay was performed on 34 post vaccination samples. There was a strong correlation between live virus neutralization titer (NT), titers of anti-WT-spike IgG and VOC specific surrogate neutralization antibody levels (Table S2).

### 3.3. T-Cell Responses

**Figure 3:**
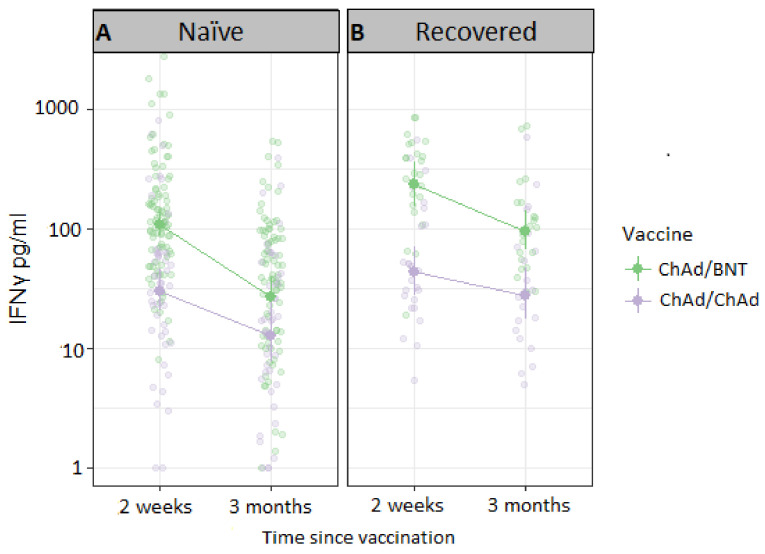
Concentration of background-adjusted interferon-gamma (IFN-γ) levels after whole-blood stimulation with 16 SARS-CoV-2 specific peptides in serial sampling at 2 weeks (14–21 days) and 3 months (75–105 days) after vaccine in (**A**) SARS-CoV-2 naïve (ChAd/BNT *n* = 92, ChAd/ChAd *n* = 48) and (**B**) SARS-CoV-2 recovered (ChAd/BNT *n* = 25, ChAd/ChAd *n* = 25) participants. IFN-γ was measured by ELISA in the supernatant. Transparent circles represent individual samples, filled circles represent geometric mean with 95% CI. IFN-γ; Interferon-γ, ChAd; ChAdOx1 nCoV-19 vaccine, BNT; BNT162b2 mRNA vaccine.

SARS-CoV-2 specific T-cell memory responses were assessed based on IFN-γ release after stimulation of fresh whole blood with a SARS-CoV-2-specific peptide pool (Figure 3). When adjusted for age, sex, vaccine dose interval and time between second vaccine dose and sampling, IFN-γ levels were 2.8-fold higher 2 weeks after ChAd/BNT, compared to after ChAd/ChAd vaccination (*p* < 0.001). At 3 months post second vaccine dose, a comparatively larger decline of IFN-γ responses was noted among recipients of heterologous ChAd/BNT, resulting in a no longer significant difference between the regimens (*p* = 0.18).

Importantly, participants that had been SARS-CoV-2 infected prior to vaccination showed higher peak IFN-γ responses 2 weeks post vaccination compared to naïve participants irrespective of vaccine regimen (2-fold higher after both ChAd/BNT (*p* = 0.028) and ChAd/ChAd (*p* = 0.058)). Differences were retained at three months post vaccine (2.5-fold higher after both ChAd/BNT (*p* < 0.001) and ChAd/ChAd (*p* = 0.003), although without a significant difference between regimens (*p* = 0.28).

There were no significant dose interval dependent effects on IFN-γ levels in any of the groups.

### 3.4. Reactogenicity Following Second Vaccine Dose in Heterologous ChAd/BNT and Homologous ChAd/ChAd Primary Vaccination Regimens

There were no self-reported severe adverse events following either of the vaccine regimens, but mild systemic and local events were more frequent after heterologous ChAd/BNT vaccination (62%) than after homologous ChAd (29%) (*p* < 0.001), Table S3. Notably, there were no significant differences in second dose reactogenicity between SARS-CoV-2 recovered and naïve participants in the ChAd/BNT group. Among recipients of the second ChAd dose however, SARS-CoV-2 recovered participants (*n* = 26) reported fever or pronounced illness (bed bound or sick leave) at a higher frequency (*p* < 0.001). There was a non-significant trend towards stronger humoral responses three months post vaccine among participants with pronounced illness after the second vaccine dose.

## 4. Discussion

This is, to our knowledge, to date the longest follow-up study with the aim to compare SARS-CoV-2 primary homologous adenovector vaccination to a heterologous vector-mRNA regimen. Our data show enhanced humoral responses up to 6 months post ChAd/BNT vaccination compared to homologous ChAd, and a non-inferior T cell response, providing additional support for the use of heterologous vector/mRNA primary immunization regimens.

Although both binding and neutralizing antibody titers were higher after ChAd/BNT vaccination compared to homologous ChAd, ChAd/BNT-elicited antibody titers displayed a more rapid decline, owing to a shorter half-life, as compared to homologous ChAd elicited antibody titers in participants who were SARS-CoV-2 naïve prior to vaccination. Although this may be explained by a more rapid clearance of initially higher peak antibody titers, half-life following homologous ChAd remained enhanced throughout the entire study period. It remains to be investigated if there is a vaccine dependent difference in durability of the humoral response.

The initially inferior humoral and cellular immune responses after homologous ChAd compared to heterologous ChAd/BNT vaccination reported here are in line with previous reports by us and others [5,6,31]. The reasons behind this are not fully understood but competitive T cell responses directed against the adenovirus vector, which would hamper the SARS-CoV-2 spike-specific immune responses, could be one explanation [31]. Antibodies against the adeno-vector induced by the first ChAd dose could also contribute to reduced antigen exposure after the second vaccine dose. Both these factors may influence the peak SARS-CoV-2 specific T-cell responses 2 weeks post second ChAd dose, but do not seem to affect the durability of responses evoked. Over time, the adjusted differences in SARS-CoV-2 T cell responses between the vaccine regimens balanced out and were at three months post vaccination no longer significant.

Both vaccine regimens elicited antibodies capable of inhibiting WT spike-ACE2 binding (surrogate neutralization) which remained detectable 3 months after vaccination. There were, however, large inter-individual variations in neutralizing antibody titers against the four VOCs in SARS-CoV-2 naïve participants, particularly after homologous ChAd. In contrast, this variation was much less pronounced among SARS-CoV-2 recovered participants, supporting a more robust humoral response in vaccinated SARS-CoV-2 recovered individuals [32].

As expected [27,33], SARS-CoV-2 infection prior to vaccination was associated with enhanced humoral and cellular responses following both vaccine regimens. Of note, in SARS-CoV-2 recovered participants, the more moderate decrease in antibody titers seen after homologous ChAd as compared to after heterologous ChAd/BNT seemed to override the initially stronger response evoked by heterologous ChAd/BNT, suggesting similar humoral responses several months post vaccination regardless of vaccine regimen if SARS-CoV-2 infection has occurred prior to vaccination.

This study is limited by the observational study design and the female dominance of the study cohort. Furthermore, we expect the longer dose interval in the ChAd/BNT group to enhance, but not fully explain, the stronger humoral response seen in this group.

## 5. Conclusions

Taken together, our findings provide further support for the use of heterologous primary SARS-CoV-2 immunization with ChAd and BNT. The regimen is tolerable and results in durable immune responses. Despite an initial rapid decline in titers, heterologous vaccination induced antibody titers and T-cell responses that remain high over time compared to those evoked by homologous ChAd vaccination. Robust and durable immune responses to heterologous vector/mRNA immunization is of great importance to mitigate consequences of variations in vaccine supply, to avoid concerns related to vector immunity and to meet updated mRNA-vaccines.

## Figures and Tables

**Table 1:** Demographics, vaccine dose interval and occurrence of SARS-CoV-2 infection prior to vaccination. BNT; BNT162b2 mRNA vaccine, ChAd; ChAdOx1 nCoV-19 vaccine, IQR; interquartile range. Age and dose interval are presented as median (interquartile range).

	BNT/BNT *n* = 210	ChAd/BNT *n* = 241	ChAd/ChAd *n* = 113
Age (IQR)	50 (40–58)	48 (39–56)	50 (39–56)
Sex			
Female	180 (86%)	211 (88%)	99 (88%)
Male	30 (14%)	30 (12%)	14 (12%)
Dose interval, days (IQR)	21 (21–28)	91 (86–94)	83.0 (77–87)
Infection prior to vaccine			
Naïve	210 (100%)	174 (72%)	73 (65%)
Recovered	0	67 (28%)	40 (35%)

**Table 2:** Estimated half-life at different sampling points after both vaccine regimens in participants either SARS-CoV-2 recovered or naïve prior to vaccination. ChAd; ChAdOx1 nCoV-19 vaccine, BNT; BNT162b2 mRNA vaccine.

	2 Weeks to 3 Months	3 to 6 Months	2 Weeks to 6 Months
Naïve			
ChAd/BNT	1.3 months (1.2–1.4)	2.7 months (2.2–3.4)	1.9 months (1.7–2.1)
ChAd/ChAd	2.5 months (2.2–3)	3.7 months (2.9–5.1)	3.1 months (2.8–3.6)
Recovered			
ChAd/BNT	1.6 months (1.4–1.9)	4.7 months (3–10.6)	2.7 months (2.3–3.3)
ChAd/ChAd	2 months (1.7–2.4)	9.8 months (5.1–129.7)	3.8 months (3.1–4.7)

## Data Availability

Not applicable.

## Supplementary Materials

The following supporting information can be downloaded at: https://www.mdpi.com/article/10.3390/vaccines10030359/s1, Figure S1. Study cohort and sampling time points. Figure S2. Surrogate virus neutralization GMTs against variants of concern 3 months after ChAd/BNT (*n* = 213, 64 SARS-CoV-2 recovered), ChAd/ChAd (*n* = 85, 29 SARS-CoV-2 recovered) and BNT *n* = 210 (0 recovered) primary vaccination. Table S1. Vaccine regimen and anti-spike-IgG levels at 1, 3, 5 and 6 months after vaccine in SARS-CoV-2 recovered and naïve participants, effect per group as factors with naïve ChAd/ChAd participants as the reference group. Table S2. Correlations between binding antibodies, neutralizing antibodies (as determined by live-virus microneutralization) and surrogate virus neutralization (as determined by ACE2-spike competitive assay). Table S3. Frequency of solicited adverse events after second dose vaccine (ChAd or BNT). ChAd; ChAdOx1 nCoV-19 vaccine, BNT; BNT162b2 mRNA vaccine.
