# Supplementary material for: Duration of SARS-CoV-2 Immune Responses Up to Six Months Following Homologous or Heterologous Primary Immunization with ChAdOx1 nCoV-19 and BNT162b2 mRNA Vaccines
Source: Vaccines (Basel). 2022 Feb 24;10(3):359. doi: 10.3390/vaccines10030359 (PMC8953845; doi:10.3390/vaccines10030359)
Supplement: Supplementary file 1 [file vaccines-10-00359-s001.zip › vaccines-1603013-supplementary.pdf]

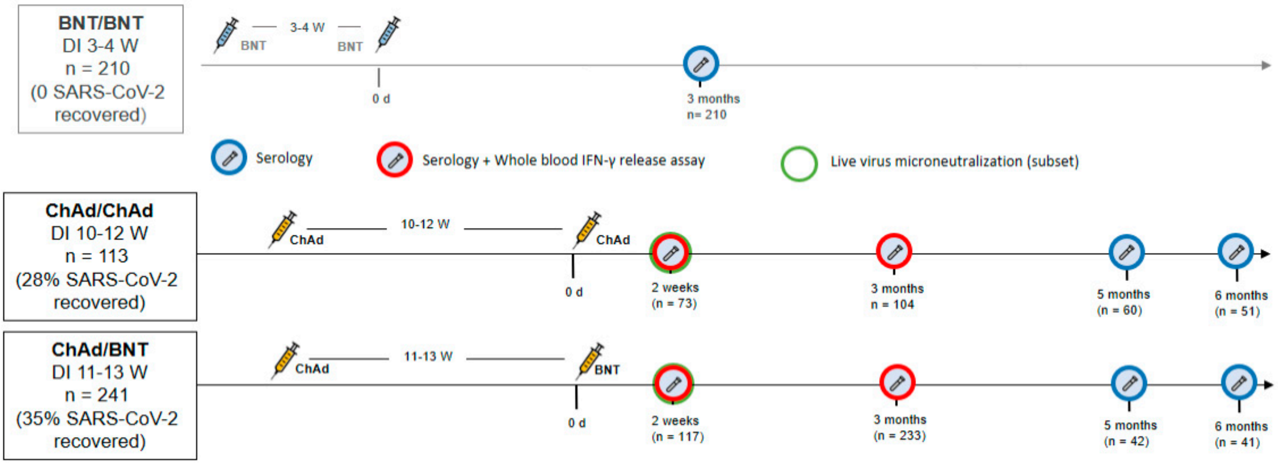

Figure S1. Study cohort and sampling time points. ChAd; ChAdOx1 nCoV-19 vaccine, BNT; BNT162b2 mRNA vaccine, DI; dose interval, W; weeks, IFN-γ; interferon gamma.

Table S1. Vaccine regimen and anti-spike-IgG levels at 1, 3, 5 and 6 months after vaccine in SARS-CoV-2 recovered and naïve participants, effect per group as factors with naïve ChAd/ChAd participants as the reference group. ChAd; ChAdOx1 nCoV-19 vaccine, BNT; BNT162b2 mRNA vaccine.

Vaccine	2 weeks	3 months	5 months	6 months
Naïve				
ChAd/ChAd	1.0 (ref)	1.0 (ref)	1.0 (ref)	1.0 (ref)
ChAd/BNT	6.0 (4.8–7.6)	3.5 (2.8–4.5)	2.9 (2.3–3.7)	2.9 (2.1–3.8)
Recovered				
ChAd/ChAd	5.2 (3.7–7.2)	4.4 (3.2–6.1)	5.3 (3.8–7.2)	6.2 (4.4–8.9)
ChAd/BNT	17.6 (13.4–23.1)	13.2 (10.2–17.0)	13.5 (10.2–17.8)	14.8 (10.5–20.8)

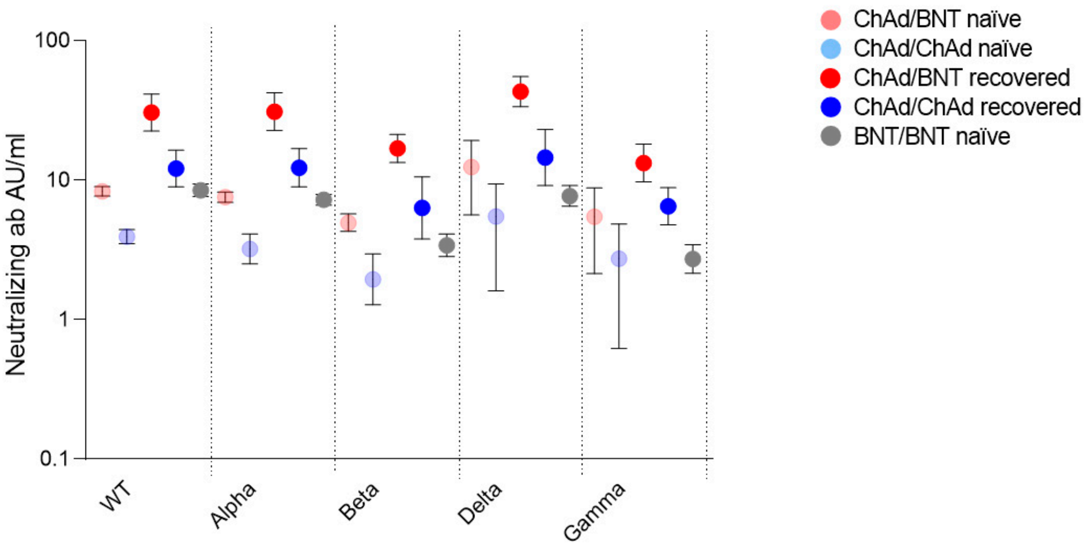

Figure S2. Surrogate virus neutralization GMTs against variants of concern 3 months after ChAd/BNT (n = 213, 64 SARS-CoV-2 recovered), ChAd/ChAd (n = 85, 29 SARS-CoV-2 recovered) and BNT n = 210 (0 recovered) primary vaccination. ChAd; ChAdOx1 nCoV-19 vaccine, BNT; BNT162b2 mRNA vaccine, Ab; antibodies, AU; arbitrary units, naïve; SARS-CoV-2 naïve prior to vaccination, recovered; SARS-CoV-2 recovered prior to vaccination.

Table S2. Correlations between binding antibodies, neutralizing antibodies (as determined by live-virus microneutralization) and surrogate virus neutralization (as determined by ACE2-spike competitive assay). WT; SARS CoV-2 wild type variant.

Correlation of assays	Spearman r (95% CI)
Delta live-virus neutralization/Delta surrogate neutralization	0.89 (0.8–0.95)
Delta live-virus neutralization/anti-WT-spike IgG	0.92 (0.84–0.96)
WT live-virus neutralization/WT surrogate neutralization	0.90 (0.80–0.95)
WT live-virus neutralization/ anti-WT-spike IgG	0.86 (0.740.93)

Table S3. Frequency of solicited adverse events after second dose vaccine (ChAd or BNT). ChAd; ChAdOx1 nCoV-19 vaccine, BNT; BNT162b2 mRNA vaccine.

	ChAd/ChAd (n = 79, 26 SARS-CoV-2 recovered)	ChAd/BNT (n = 117, 25 SARS-CoV-2 recovered)	p
Any adverse event			
No	57 (72%)	44 (38%)	< 0.001
Yes	22 (28%)	73 (62%)	
Fever >38°C			
No	77 (97%)	102 (87%)	< 0.05
Yes	2 (3%)	15 (13%)	
Local reaction			
No	70 (89%)	79 (68%)	< 0.001
Yes	9 (11%)	38 (32%)	
Mild general symptoms			
No	72 (91%)	92 (79%)	< 0.05
Yes	7 (9%)	25 (21%)	
Pronounced general symptoms			
No	74 (93%)	93 (79%)	< 0.05
Yes	5 (7%)	24 (21%)	
